# Contemporary Parallel Diversification, Antipredator Adaptations and Phenotypic Integration in an Aquatic Isopod

**DOI:** 10.1371/journal.pone.0006173

**Published:** 2009-07-09

**Authors:** Fabrice Eroukhmanoff, Erik I. Svensson

**Affiliations:** Section for Animal Ecology, Lund University, Lund, Sweden; Duke University, United States of America

## Abstract

It is increasingly being recognized that predation can be a strong diversifying agent promoting ecological divergence. Adaptations against different predatory regimes can emerge over short periods of time and include many different traits. We studied antipredator adaptations in two ecotypes of an isopod (*Asellus aquaticus*) that have, diverged in parallel in two Swedish lakes over the last two decades. We quantified differences in escape speed, morphology and behavior for isopods from different ecotypes present in these lakes. Isopods from the source habitat (reed) coexist with mainly invertebrate predators. They are more stream-profiled and have higher escape speeds than isopods in the newly colonized stonewort habitat, which has higher density of fish predators. Stonewort isopods also show more cautious behaviors and had higher levels of phenotypic integration between coloration and morphological traits than the reed isopods. Colonization of a novel habitat with a different predation regime has thus strengthened the correlations between pigmentation and morphology and weakened escape performance. The strong signature of parallelism for these phenotypic traits indicates that divergence is likely to be adaptive and is likely to have been driven by differences in predatory regimes. Furthermore, our results indicate that physical performance, behavior and morphology can change rapidly and in concert as new habitats are colonized.

## Introduction

An increasing number of workers now recognize that predation can play an important role in evolutionary diversification, and that resource competition is not the only force that can drive adaptive divergence [1]–[4]. Previous work in this area have focused on diverging populations or species that experience similar predation pressures but in heterogeneous habitats [5] or those that involve different predatory regimes (high vs. low) [6] or different predators [7], [9]. Prey populations colonizing new environments should quickly become locally adapted, otherwise low population fitness will eventually lead to extinction of the novel populations [3]. However, examples of contemporary evolution in the context of predator-mediated divergence remain scarce [3] and often only document a change in a single population, which precludes broader generalizations of the observed patterns. Cases of rapid parallel evolution, especially when involving different sets of traits, provide unique opportunities to investigate how adaptive divergence is constrained in its early stages by trade-offs and historical contingencies, and how it might affect overall phenotypic integration in prey organisms.

Antipredator adaptations can be morphological, such as when prey evolve cryptic or aposematic pigmentation [10]–[13], behavioral (e.g. reduction in the frequency of bold behaviors) [7], [14] or effect aspects of physical performance (e.g. to increase ability to escape from predators) [6], [9], [15]. Antipredator adaptations are often also correlated with different life history traits [16] which might lead to trade-offs between different traits from which adaptive constraints might arise [6]. Antipredator adaptations can also function as sexual isolation characters and species recognition cues, and these adaptations might then interfere with mate preference divergence and indirectly promote reproductive isolation [17]. The ecological origin and fitness consequences of such interactions between antipredator adaptations and other phenotypic traits are therefore of central interest to evolutionary ecologists.

**Figure 1:**
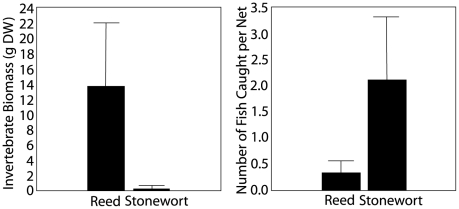
Types of potential predators present in the source reed ecotypes and in the new stonewort ecotypes. Invertebrates such as dragonfly larvae or beetle larvae are more common in the reed, whereas a number of fish species such as perch are more common in the stonewort. Modified from Wagner & Hansson (1998).

In this study, we investigate potential antipredator adaptations that have emerged following the parallel emergence of two different ecotypes of a freshwater isopod (*Asellus aquaticus*) in lakes in southern Sweden. During the past two decades, a recently emerged habitat (stonewort; *Chara tomentosum*) has been colonized by isopods in southern Swedish lakes. The isopods colonized the stonewort from a source habitat consisting of reed *Phragmites australis* [18]. In the reed habitat, the main predators are invertebrates such as damselfly and dragonfly larvae while visually hunting predators like fish are uncommon [19]–[20] (Fig. 1). In contrast, in the novel stonewort habitat, various fish species (such as perch, *Perca fluviatilis*) are common, but invertebrate predators are almost entirely absent [20] (Fig. 1). These qualitative differences in the types and numbers of different predators are the most likely causal factors behind the parallel phenotypic divergence in two of the most intensively studied lakes [18].

In a recent study, we have showed that in these lakes, stonewort ecotype isopods were smaller in body size and had lighter body pigmentation [21], and these phenotypic changes are remarkably parallel with only a minor role for historical contingency [21]. These morphological changes were also accompanied by differences in mating behaviors, with the stonewort ecotype showing lower mating propensity than the reed ecotype [21]. Both morphology and mating behavior have thus diverged in parallel in both lakes following invasion of the novel stonewort habitat. Molecular genetic analyses using nuclear (AFLP) and mtDNA markers revealed that the stonewort ecotypes in the two lakes have independent evolutionary origins, strongly suggesting that this is indeed a case of parallel evolution [22], on an extremely small temporal and spatial scale. Cases of contemporary evolution in the context of predation often describe changes occurring in one population [23] but until recently, few studies had shown how entirely different sets of traits such as physical performance, behavior and/or morphology could change in parallel [24] over a short temporal scale [3].

Here we extend our previous work by quantifying parallel changes in body shape, escape speed, and differences in exploratory behavior in one of the two study lakes. Our aim was to investigate if and how these two different ecotypes have diverged in their putative antipredator adaptations. We show that the reed ecotype have higher escape speed and have a higher stamina, presumably because its overall body shape generates less drag during swimming. In contrast, the isopods from the recently colonized stonewort habitat have a higher level of phenotypic integration in morphology due to more strengthened correlations between pigmentation and body size. Stronger phenotypic integration [25] between shape-related and pigmentation-related traits could presumably be a result of selection for smaller and lighter pigmented individuals, which should increase crypsis in the stonewort habitat. In addition, we have previously documented lower mating propensity in the stonewort ecotypes [21]. Together with a the lower exploratory activity we report in this study from stonewort individuals from one lake, these behavioral differences indicate that more prudent behaviors are favored in the novel stonewort habitat, possibly in order to avoid detection from predators.

At this point we should add some clarifying caveats of our study. First, we do not know to what extent these phenotypic differences between ecotypes are heritable, and plasticity could possibly have played some role in ecotype differentiation. Second, we have not clearly established a clear connection between the adaptive character of these phenotypic changes and predation, since we have not demonstrated that these changes had an effect on survival. However, some of the traits investigated here, such as escape speed, are clearly likely to be important during predator encounters. Indeed, these adaptations are habitat-specific, but they could also have changed as a response to different foraging pressures or other ecological conditions differing between habitats [26].

## Methods

### Study organism: natural history and ecology

*Asellus aquaticus* is a freshwater isopod that is widespread in lakes, ponds and slow-flowing rivers [18]. Populations *of A. aquaticus* occupy various habitats in lakes, but are concentrated in reed stands (*Phragmites australis*) where they feed on decaying leaves [18]. Two shallow Swedish lakes, Lake Krankesjön (55°42′N, 13°28′E) and Lake Tåkern (58°21′N, 14°50′E), have in the past twenty years (starting in 1987 in Lake Krankesjön and in 2000 for Lake Tåkern) experienced dramatic ecological shifts from a phytoplankton dominant state towards an macrophyte dominated state [27]–[28]. These ecological shifts resulted in the colonization of sediment lake bottoms in the limnetic zone by stonewort (*Chara tomentosa*). Following the establishment of stonewort, isopods also colonized the novel habitat. In both Lake Tåkern and Lake Krankesjön, as in five other lakes, habitat-specific changes in pigmentation were observed following colonization: isopods became brighter in the stonewort stands compared to the darker ancestral populations in the reed, and these pigmentation differences are largely heritable [18]. It is important to note that there is evidence that, at least in Lake Krankesjön, these phenotypic changes occurred after colonization of the stonewort, since when isopods were initially sampled shortly after the stonewort started to expand (in 1987) they were phenotypically similar to reed individuals [18]. Therefore, it is highly probable that these habitat-specific phenotypes are the result of a diversification event which began about twenty years ago. The substrate in the reed consists of organic detritus that form a black background, whereas the stonewort habitat consists of light green vegetation growing above a light grey mineral substrate.

Local adaptation in isopod pigmentation is likely a result of divergent selection pressures caused by the different visual backgrounds and predator faunas in the two habitats [18], [29], two factors which commonly drive the evolutionary dynamics of predator-prey communities in many aquatic habitats [30]. Similar locally cryptic and adaptive color differentiation have been documented in seven other south Swedish lakes, the parallel changes observed in Lake Tåkern and Lake Krankesjön are not unique and not restricted to these two particular lakes [10]. Fish are highly efficient predators on invertebrates [30], and *A. aquaticus* is a common prey item on this type of habitats [19]. These transitions to the new limnetic habitat consisting of submerged vegetation was beneficial for some fish species, such as perch (*Perca fluviatilis*), whose populations grew in a correlated fashion with the expansion of the stonewort [29] (Fig. 1). Predation from visually hunting fish is thus likely to be much more intense in the stonewort habitat than in the reed, due to higher densities of perch in the novel habitat [20] (Fig. 1). This ecological difference between the two habitats has been suggested to select for smaller, brighter isopods in the stonewort habitat. In contrast, in the reed habitat, invertebrate “sit-and-wait”- predators that rely primarily on tactile cues (i.e. dragonfly and damselfly larvae) are the main threat towards the isopods [10], [20].

### Experiments on escape speed and endurance

In spring 2007, a total of 20 males from each ecotype (reed and stonewort) and each lake (Lake Tåkern and Lake Krankesjön) were collected in the field (N = 80 males in total). Isopods were captured with hand-nets and were kept in common containers at the same temperature at 21°C and with abundant food for two days to remove any possible habituation effects on the experimental trials. The isopods were then separated into “large” and “small” size classes (10 in each class) for each habitat, because the two ecotypes differ in size [18]. These size classes were above or below .95 cm for the reed ecotypes, and above or bellow .75 cm for the stonewort ecotype. The categorization of sizes also enabled us to investigate the role of size as a confounding factor in the analysis of escape speed, and to determine if differences between ecotypes in performance traits were confounded by differences in size.

Escape speed trials were performed by placing each individual in a Petri dish filled with water. Individuals were then constantly poked with a stick to simulate a predator attack for 30 seconds and the circular distance they moved during this time was measured and translated into a linear distance. By dividing this linear distance with the time (30 s) we obtained an average speed for each individual isopod. After 5 minutes of rest, we performed a second experiment trial aimed to quantify speed and performance loss. The difference between the linear distance moved during the first trial and the second trial was used as a measure of physiological endurance. Differences between ecotypes were first analyzed with repeated-measures ANOVA, with lake and habitat as factors, and their interaction with the repeat (individual). This interaction provides a statistical test of initial speed and performance loss within individual isopods, and relates this performance loss to habitat and lake of origin. Significant interactions between the factors and the repeat (i. e., repeat*habitat, repeat*lake) indicate significant effect of either habitat or lake on performance loss. In contrast, significant main effects (habitat and lake, respectively) indicate an overall difference in isopod vigor between habitats and/or lakes. Subsequently, we also tested for the possible confounding effects of individual body size, by including size class as a categorical predictor alongside with ecotype and lake of origin.

Finally, to test for a physiological trade-off between speed and endurance, we performed linear regressions within each ecotype and used an ANCOVA for speed in the second trial, with speed in the first trial as a continuous predictor and lake and ecotype as categorical predictors, as well as all the possible double interactions between these three terms. A trade-off between speed in the second and in that in the first trail would manifest itself as a negative effect of speed of the first trial in this model. If this trade-off differs between ecotypes, it would manifest itself as a significant interaction between speed in first trial and habitat on the speed in the second trial. All statistical tests in this study were performed using the software STATISTICA [31].

### Shape analysis: geometric morphometrics

Digital photographs were taken from twenty additional males from each population (two ecotypes in both lakes; N = 80).Ten homologous landmarks were identified and subsequently used (Figure S1) in geometric-morphometric techniques, as described by [32]. The shape differences obtained from these landmarks will be independent of isometric shape differences [33] and will reflect a consensus configuration [32]. Using the software TPS [32] (the thin-plate spline method), one can then assess deformations from the consensus landmark configuration and perform statistical shape analyses on relative warps (RWA). We analyzed the weight matrix of each population using a MANCOVA (with size as a covariate to remove isometric size effects, since the reed ecotypes are significantly larger than the stonewort ecotypes). We subsequently tested for differences between ecotypes and lakes. The first two canonical variates obtained from the RWA weight matrix were computed using TPS module [32].The generated shape deformations along these two relative warp axes were used to visualize ecotype differences.

To calculate the effects of shape on hydrodynamic profile and on potential speed, we measured the angle (α) between the tip of the head and the extremities of the first segment (which is henceforth called the “head angle” for simplicity). This angle gives an estimate of the total amount of potential friction that is created by the anterior morphology of the isopods as they move through the water. By comparing the average angle between ecotypes and lakes, we calculated the potential hydrodynamic consequences of the shape differences between the populations for a simplified drag model [15]. We then calculated the different drag coefficients for each of the ecotypes and the expected drag force according to the following equations [15]:
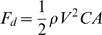
(1)where *F_d_* is the force of drag, ρ is the density of water, *V* is the velocity, *C* is the coefficient of drag, and *A* the projected surface. The drag coefficient can be obtained:
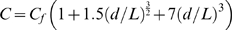
(2)where *C_f_* is the frictional drag coefficient, *d* is the mean value of the maximum width and depth and *L* the total length. For intermediate conditions between turbulent and laminar flows, *C_f_* can be calculated using:
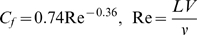
(3)where Re is the Reynolds number and υ is the kinematic viscosity of water.

Combining and rearranging equations 1–3, the ratio between the expected drag forces experienced by individuals of each ecotype at equal speed is equal to:
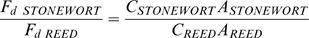
(4)By calculating replacing *A* with a simplified upper morphology model (a right triangular isosceles prism) including the head angle α,Eq. 4 simplifies to:
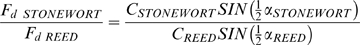
(5)From Eq. 5, one can thus estimate the supplementary amount of drag that is experienced by the average-shaped stonewort isopods if they would move at the same speed as the reed individuals.

### Exploratory behavior

We captured a total of 150 isopods from both ecotypes in Lake Krankesjön which were then acclimated in the laboratory for 2 days, at a temperature of 21°C. During this period, isopods were feeding on their original substrate that was sampled upon capture. Animals were then randomly divided into groups of 50 individuals for each trial (6 trials in total) and placed in a 30 cm high aquarium (30 cm*70 cm), containing the substrate of their native habitat (stonewort shoots or decaying reed leaves) in one end, and the substrate of the other lake habitat on the other end. Isopods were always placed in their original substrate. At distance of 40 cm separated the substrates at either end of the aquarium. After 24 hours, a census was made within each substrate to determine the proportion of individuals that moved between substrates. We used a General Linearized Model (GLZ) with a binomial distribution, with ecotype and trial as categorical factors to assess any differences between ecotypes in exploratory behavior, i. e. the propensity to disperse and to forage on another substrate than the native substrate. Trial was included as a factor to control for possible differences in exploratory behavior between sessions that could potentially cause statistical non-independence (it turned out that trial was not significant, however, see Results).

### Visualizing and estimating phenotypic integration

To estimate phenotypic integration in morphology and pigmentation in the different ecotypes, we analyzed data from a total of 805 individual isopods that were photographed and measured for an earlier study [21]. Conditional independence for 4 morphological traits (length (L), width at segment 1, 4 and 7 (W1, W4 and W7)) and 3 pigmentation traits (H, a color parameter, S, a saturation parameter and V, a brightness parameter) were estimated from partial inverse correlation matrices [34]. We subsequently tested for edge exclusion deviance *D* (exclusion of the near-zero elements of the inverse correlation matrix) using the formula:
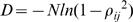
(6)W where *N* is the sample size, and *ρ_ij_^2^* is the partial correlation coefficient between trait i and j with all other elements held constant [34]. Each edge exclusion deviance was then tested against the χ^2^-distribution with one degree of freedom and all edges with deviance greater than 3.84 were rejected, reflecting a 5% significance level on the χ^2^-distribution, with one degree of freedom [34].

From the data on edge exclusion deviance, we constructed conditional independence graphs, as described by Magwene [34]. Conditional independence graphs show relationships (edges) between traits (vertices) that remain when underlying, shared correlations with other traits have been removed [34]. Conditional independence graphs will thus reveal how independent or “embedded” a particular trait is [35]. Conditional independence graphs were constructed for the two ecotypes in each lake, generating a total of four different graphs. The aim of conditional independence graphs is to visualize the patterns of phenotypic integration, to assess differences and parallelism in integration levels between habitats and lakes. To quantify these differences, we estimated two parameters from each graphs. First, we calculated the average connectivity per trait for each population. Then we compared habitats within lakes using a General Linear Model (GLM) with ecotype, lake as categorical predictors and their interaction, to estimate differences in connectivity between habitats. Second, we compared the modular structure of the graphs to identify potential biological modules: one involving shape morphology (henceforth called “morphological module” for simplicity and including L, W1, W4 and W7) and another one involving pigmentation (henceforth called “pigmentation module” and including H, S and V). We then used the formula below to calculate an index of modularity (*I_M_*) for each graph [36]:
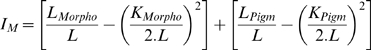
(7)where *L* is the number of edges in the entire graph, *L_Morpho_* the number of edges within the morphological module, *K_Morpho_* the sum of the number of traits each trait is connected to within the morphological module, *L_Pigm_* the number of edges within the pigmentation module, and *K_Pigm_* the sum of the number of traits each trait is connected to within the pigmentation module. This index varies between 0 (no modular structure) and 0.5 (the inverse of the number of modules in the graph).

## Results

### Escape speed and endurance

**Figure 2 pone-0006173-g002:**
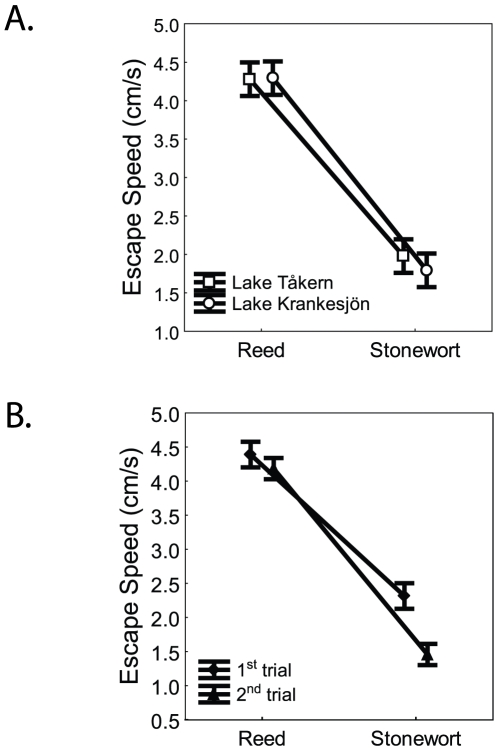
Escape speed (in cm/s) and endurance differences between lakes and habitats (ecotypes). Differences between habitats across both lakes were significant for both initial speed (A) and endurance (Both lakes pooled) (B), as revealed by a repeated-measures ANOVA (Table 1). Differences between lakes were not significant; i. e.ecotypes were more similar to the same ecotype in the other lake than to the different ecotype in the same lake. However, reed individuals had higher endurance and did not lose speed after the first trial (Tukey Post-Hoc test: *P* = .0503), whereas stonewort individuals had a significantly lower speed at the second trial (Tukey Post-Hoc test: *P*<.001).

In Fig. 2, we show the average escape speed for each lake the changes between the first and second trial. The repeated-measures ANOVA for escape speed revealed strong parallelism in speed divergence within ecotypes across lakes (Table 1; Fig. 2A). There was a highly significant and strong main effect of habitat but no main effect of lake, indicating only a weak historical signal of lake origin (Table 1). Thus, ‘ecology’ (habitat) had an overriding effect on ‘history’ (lake) in explaining differences in escape speed in these isopods. Moreover, endurance also differed between the different ecotypes, again with weak and non-significant effects of lake (Table 2, Fig. 2B). Stonewort isopods were slower than reed isotopes in the first trial, and their speed also decreased more the second trial. Thus, stonewort isopods had both lower initial speed and endurance (Table 2, Fig. 2B). This difference in speed loss between the different ecotypes expressed itself as a significant interaction between the repeat (speed at first and second trial) and ecotype (Tables 1 and 2B).

**Table 1:** Repeated Measures ANOVA of escape speed for lakes and ecotypes.

Category	Effect	df	MS	F_1,76_	*P*
**Between Subject**					
	**Lake**	1	0.295	616	0.434
	**Ecotype**	1	230.52	481.8	<0.001
	**Size**	1	6.24	13.041	<0.001
	**Lake×Ecotype**	1	0.397	0.829	0.365
**Within Subject**					
	**Repeat**	1	11.34	93.01	<0.001
	**Repeat×Lake**	1	0.038	0.314	0.577
	**Repeat×Ecotype**	1	4.26	34.97	<0.001
	**Repeat×Lake×Ecotype**	1	0.136	1.12	0.293

**Table 2 pone-0006173-t002:** Escape speed in cm/s in the four different study populations.

Lake	Ecotype	Escape Speed 1^st^ trial (±SE)	Escape Speed at 2^nd^ trial (±SE)	Performance loss (%,±SE)
**Krankesjön**	**Reed**	4.382 (±.143)	4.204 (±.147)	3.611 (±2.380)
**Tåkern**	**Reed**	4.396 (±.145)	4.162 (±.136)	4.974 (±1.760)
**Krankesjön**	**Stonewort**	2.267 (±.142)	1.319 (±.081)	37.91 (±5.388)
**Tåkern**	**Stonewort**	2.363 (±.112)	1.594 (±125)	31.93 (±4.707)

NOTE. – Reed isopods in both lakes were faster in both the 1^st^ and 2^nd^ trial, and they also had higher endurance (lower performance loss between the 1^st^ and 2^nd^ trial) than the stonewort isopods (Figs. 1–3; Table 1).

**Figure 3:**
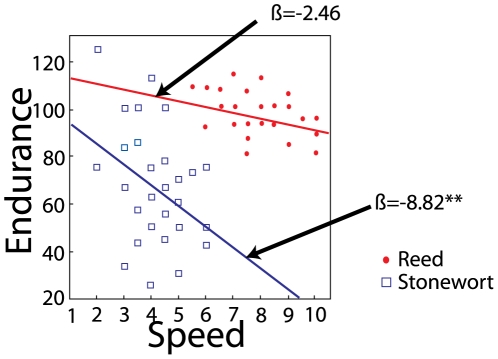
Trade-off between speed and endurance (the percentage of speed maintained during the second speed trial) in both ecotypes. Linear regression between endurance and speed for individuals from the reed and the stonewort. Both regression coefficients are negative, although there is no significant correlation between speed and endurance in the reed (Stonewort: r^2^ = 0.163, *P* = 0.0098, β = −8.82; Reed: r^2^ = 0.0946, *P* = 0.0536, β = −2.46) and the slopes differ significantly between ecotypes, being steeper among stonewort isopods (GLM for endurance: Ecotype*Speed interaction: F_1,74_ = 4.003, *P* = 0.0491).

We found evidence for a trade-off (negative relationship) between endurance and speed in Fig. 3. In the stonewort ecotypes, we found a negative relationship between endurance and speed (r^2^ = 0.163, *P* = 0.0098, β = −8.82) but only a marginal decline in speed in the reed ecotypes (r^2^ = 0.0946, *P* = 0.054, β = −2.46). We investigated this further using an ANCOVA with endurance as the dependent variable, and escape speed in first trial, lake and ecotype and their interactions as independent variables. There was a significant effect of speed on endurance: F_1,74_ = 12.398, *P*<0.01, meaning that across both ecotypes, faster individuals had less endurance, i. e. a trade-off. However, this trade-off also differed between the ecotypes, as revealed by a significant interaction between initial speed and ecotype (F_1,74_ = 4.003, *P*<0.0491). The trade-off was more pronounced in the stonewort ecotype than in the reed ecotype, as revealed by a steeper negative slope in Fig. 3. Hence, stonewort isopods paid a higher cost, in terms of endurance, for high initial speed. The trade-off was thus habitat-specific (Fig. 3).

### Shape analysis and hydrodynamic consequences

**Figure 4:**
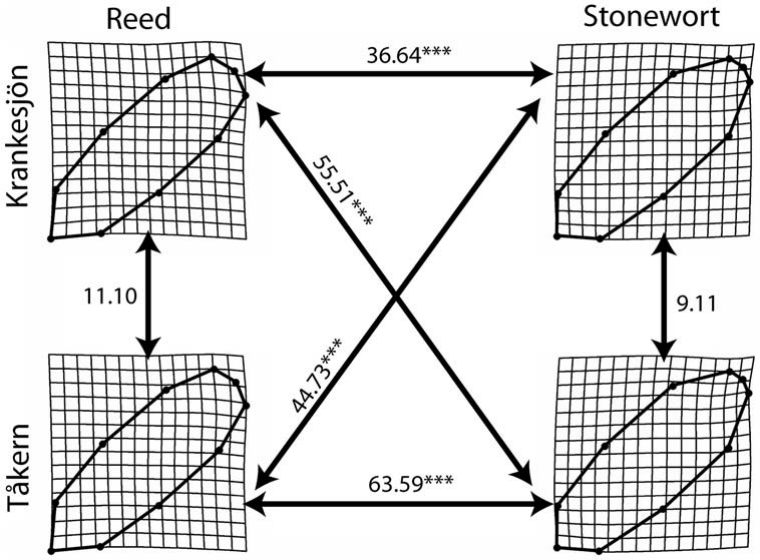
A visualization of shape divergence between ecotypes and lakes. Thin-plate splines transformations represent the average shape deformation for each population from the consensus shape. Reed isopods have a different hydrodynamic profile shape (sharper anterior body and wider posterior body). The arrows indicate the Euclidian distance between two population average shapes calculated from a MANCOVA (Table 3). The Euclidian distances between the ecotypes are highly significant and 3 to 6 times greater than the distances within ecotypes, even if these isopods would come from different lakes. There are thus no significant differences in shape between lakes within the same ecotype.

Thin-plate splines transformations for each ecotype and lake are shown in Fig. 4. Using a MANCOVA (with size as a covariate) on the weight matrix from the partial warps (Table 3), we found pronounced differences between ecotypes (habitats), but no significant differences between lakes. There were no significant effects of Lake or Lake×Ecotype on shape (Table 3), again confirming only a weak historical signature in the shape differences, as it has been shown for other traits [21]. Again, a significant habitat effect revealed strong parallelism in shape of the same ecotypes of different lakes (Table 3). To visualize this, we calculated the Euclidian distance between the four population mean shapes from the MANCOVA. The Euclidian distances between the ecotypes are significant and 3 to 6 times greater than the distances between the same ecotypes (Fig. 4). Thus, although the isopods of the same ecotype come from different lakes and are genetically and geographically closer to the alternative ecotypes within their own lakes, there were significant differences between ecotypes but not between isopods that come from different lakes but belong to the same ecotypes (Fig. 4).

**Table 3 pone-0006173-t003:** MANCOVA on the weight matrix from the relative-warps analysis based on 16 dependent variables.

Source	Wilks Λ	F_1,35_	*P*
**Lake**	0.46	1.469	0.206
**Ecotype**	0.075	15.41	<0.001
**Size**	0.442	1.576	0.167
**Lake×Ecotype**	0.454	1.504	0.192

NOTE. – Size is included as a covariate to remove potential isometric effects on shape.

**Figure 5:**
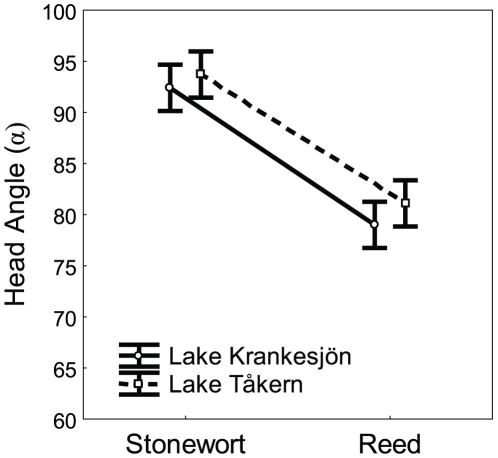
Differences in hydrodynamic profiles (penetration angle, measured as α, the angle between the front-head and the extremities of the first width segment) between isopod ecotypes. As a consequence, at equal speeds, the ratio between the forces of drag sustained by the reed individuals and the stonewort individuals is equal to 0.8522. Thus, to be able to swim at the same speed, the average stonewort isopod would have to produce a propulsion force 17.3% higher than the average the reed isopod.

Strong parallelism was also found for head angle α (Table 4). There was no effect of Lake or Lake×Ecotype on this aspect of anterior body morphology (α ) but a highly significant effect of ecotype on head angle (α) (Table 4). By combining these morphological data with equation (5), we can calculate the hydrodynamic differences between the ecotypes (Fig. 5). At equal speeds, the ratio between the forces of drag sustained by the reed individuals and the stonewort individuals is estimated to be 0.8522 (Fig. 5). Using the inverse of this ratio, we can estimate the extra amount of force (in%) that would be needed by an average stonewort isopod to swim at the same speed as an average reed isopod. This estimated extra amount of force needed in stonewort individuals to obtain the same speed as reed individuals was calculated to be 17.3%. Although other factors (e. g. internal physiological differences) could affect the ecotype differences in speed and endurance (Figs. 2–3), the extra hydrodynamic costs estimated here can partly explain these ecotype differences (Table 2).

**Table 4:** GLM for head angle (α) with lake and ecotype as fixed factors.

Source	df	SS	F_1,36_	*P*
**Lake**	1	28.9	2.33	0.136
**Ecotype**	1	1690	136	<0.001
**Lake×Ecotype**	1	1.6	0.13	0.722

### Exploratory behavior

Reed ecotype isopods had a higher propensity to leave their original substrate, and forage on the alternative substrate than had stonewort individuals (probability to explore a new substrate for reed individuals: 0.3 (SE:0.037); for stonewort individuals: 0.18 (SE:0.031)). This difference between the two ecotypes in exploratory behavior was significant (GLZ: χ(1) = 5.37; *P* = 0.021), and there was no significant replicate effect (χ(5) = 4.17; *P* = 0.124).

### Phenotypic integration

**Figure 6:**
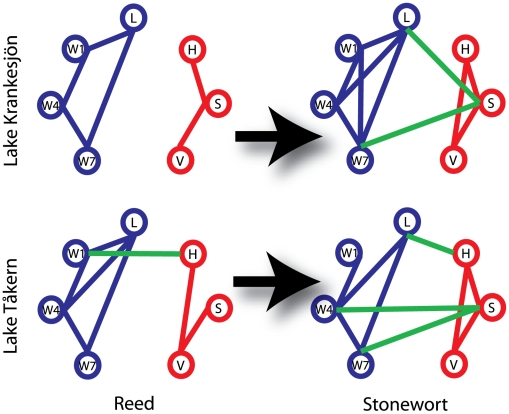
Phenotypic integration in isopod shape-related morphological traits (L: length, W1, W4, and W7: width at segment 1, 4 and 7) and pigmentation traits (H: color, S: saturation and V: brightness) for each ecotype of each lake. Each diagram is a conditional dependence graph between traits estimated from the inverse of the phenotypic correlation matrix of each population. Edges in blue represent significant connections between traits within the morphological module, edges in red connections between traits within the pigmentation module and edges in green connections between the two modules.

Conditional independence graphs for both ecotypes of each lake are shown in Fig. 6. All the depicted edges in these graphs are significant. These conditional independence graphs revealed several common patterns within ecotypes of both lakes but very little or no differences between lakes. Out of the 21 potential edges between these seven traits, we found six and eight significant edges for the reed ecotype populations in Lake Krankesjön and Tåkern, respectively, thus less than 35% of all potential edges were present in the reed ecotype. However, in the recently established populations containing the stonewort ecotype, we found 11 and 10 significant edges for Lake Krankesjön and Tåkern, respectively (Fig. 6). Thus, in the stonewort habitat ca. 50% of the potential edges were significant (Fig. 6), reflecting an overall increase in phenotypic integration in both lakes.

The average connectivity per trait was higher in the stonewort isopods than in the reed isopods, as suggested by a significant ecotype effect (F_1,24_ = 10.14; P = 0.004) (Table 5), but there was no significant lake effect (F_1,24_ = 0.207; P = 0.65) or lake*ecotype effect (F_1,24_ = 10.11.2864; P = 0.18). This again indicates a strong parallelism between lakes during ecotype diversification. These ecotype differences in phenotypic integration were not only quantitative, as shown above and in Fig. 6, but the ecotypes also differed qualitatively in their overall degree of modularity. Following the definitions for true modules outlined by Magwene (2001), we found that the isopods in one of the reed populations (Lake Krankesjön) had a pronounced modular structure, consisting of two modules, one involving the metric morphological traits (L, W1, W4 and W7) and the other involving pigmentation traits (H, S, and V (Fig. 6). The reed ecotype of the other lake (Lake Tåkern) had similar modular properties, except for one edge between H and W1 (Fig. 6). In contrast, there was no evidence of any modular structure at all in the two populations of the stonewort ecotype from the two lakes (Fig. 6). All traits in the stonewort populations were deeply connected to each other, resulting in an increased overall degree of phenotypic integration of all the traits, compared to the situation in the ancestral reed ecotype in both lakes (Fig. 6). In both lakes, the number of edges between the pigmentation traits and the morphological traits increased from the source reed ecotypes in both lakes (from zero to two edges in Lake Krankesjön, and from one to three edges in Lake Tåkern) (Fig. 6). We therefore calculated *I_M_*, the index of modularity, for each graph (Table 5). The reed ecotype from Lake Krankesjön and Lake Tåkern had higher indices of modularity (0.444 and 0.422 respectively, close to the maximal value of 0.5) than the stonewort ecotypes (0.364 and 0.270 respectively).

**Table 5:** Phenotypic integration indices in the four different isopod populations (two populations from different ecotypes in two different lakes).

Lake	Ecotype	Connectivity (±SE)	Modularity
**Krankesjön**	**Reed**	1.714 (±0.184)	0.444
**Tåkern**	**Reed**	2.286 (±0.286)	0.422
**Krankesjön**	**Stonewort**	3.143 (±0.340)	0.364
**Tåkern**	**Stonewort**	2.857 (±0.404)	0.27

NOTE. – The average connectivity per trait is significantly higher in the novel stonewort ecotypes than in the source reed ecotypes (F_1,24_ = 10.14; P = 0.004). In contrast, the index of modularity (ranging from 0 (total absence of modular structure) to 0.5 (full modular structure)) is higher in the source reed ecotype in both lakes compared to the novel stonewort ecotype.

## Discussion

It has been recently illustrated how antipredator defenses can quickly evolve as a response to predatory pressures [3], [23] and lead to rapid adaptive radiations [11]. Especially true for aquatic systems, multiple adaptations can emerge jointly in response to a specific type of predator [7], [37] or when different predator faunas occur in different habitats (our study). Two fundamental categories of defense mechanisms are avoiding detection or escaping from the predators [38]. The latter mechanism may be selected for in the reed habitat, as reed isopods are faster at escaping and have higher endurance than stonewort isopods. The morphology of the reed isopods might also have become adapted for fast locomotion since they match similar shape-related features in other aquatic animals, such as fish [6], [15]. Shapes that are wider at the posterior part of the body and have a sharper angle at the anterior part of the body are known to possess an advantageous hydrodynamic profile, which will limit drag and allow better propulsion [9], [15].

This was recently experimentally investigated and discussed thoroughly by Langerhans et al. [9], who also formulated a new ecomorphological paradigm based on a biomechanical model of swimming. These workers found a significant interaction between shape and speed between populations that differed in overall predation pressure [9]. However, the authors did not further investigate the underlying physical mechanisms which might translate shape into different speed properties. Indeed, shape could also be correlated with other underlying physical or physiological traits which might also influence speed or strength [39]. Here we have shown that the stonewort isopods have a broader anterior head angle (Fig. 5) and a thinner tail than isopods in the reed habitat. This shape changes increases the drag generated by the stonewort individuals when swimming as compared with the ancestral reed individuals. This supports the recently proposed biomechanical model by directly connecting shape to swimming performance through a model based on the properties of fluid mechanics, although in this particular instance, we are assuming a limited role for frictional drag [9], [15].

We would like to stress that head shape and its influence on fluid penetration is certainly not the only factor affecting speed. Overall physical condition, muscular mass, metabolic rate and size are other potential factors that could contribute to explain speed differences [15], [40]–[42] (Figs. 2–3). A simple biomechanical model has thus its limitations here, since to be able to swim at the same speed, the average stonewort isopod would have to produce a propulsion force 17.3% higher than the reed isopods if one considers drag as the only factor affecting speed. The speed differences between ecotypes are, however, far greater than this, because the isopods from the reed were almost twice as fast as individuals form the stonewort (Table 2). Other factors involving general physical condition and physiology could therefore also contribute to these ecotype differences. Some of these other factors could be associated with the shape differences (e. g muscle mass). Thus, the relationship between shape and physical performance are likely be more multifactorial [38] than to be explainable entirely in terms of differences in hydrodynamic profile as suggested by a recent biomechanical model [9].

Physiology and resource limitation are classical factors known to generate trade-offs between performance traits [41]. One such classical trade-off is the one between speed and stamina, which has been documented in several different animals, including lizards and frogs [40]–[41]. This trade-off is closely associated with predation risk [42] and partly reflects underlying physiological principles regarding how muscle fibers adapt differently to short and intensive or prolonged effort [40].

A difficulty in investigating trade-offs is that one cannot infer the presence of a trade-off by comparing individuals from different environmental conditions, because environments might differ in overall resource levels [44]. Here we have found that there might be overall difference in “general vigor” between ecotypes, and we have also documented a fundamental trade-off between endurance and speed within each of the isopod ecotypes (Fig. 3). According to our results, the reed isopods do not suffer from this fundamental trade-off unlike the stonewort isopods (Fig. 3), as the reed isopods are both faster and have higher endurance than the stonewort isopods (Table 2, Fig. 2). Thus, the reed isopods perform better overall than the stonewort isopods, and are able to have both high speed and high endurance. Quality differences between individuals within a population can render eventual trade-offs undetectable, and this could also be the case here [43].

The seemingly paradoxical finding above suggests an overall positive correlation between two different performance traits. The issue of habitat-differences and how they might obscure the detection of trade-offs has been extensively discussed among life-history theoreticians [43] and quantitative geneticists [44]–[45]. One conclusion from these results is that some individuals can simply perform better than others, without paying any apparent costs [44]–[45]. For instance, reed isopods could simply have a genetically or phenotypically higher overall “vigor” [43]–[45] compared to the stonewort isopods [44]–[45]. Alternatively, reed isopods might suffer from other (hidden) trade-offs, for example between escape strategy and predation risk, as reed isopods are keener on exploring new habitats. The higher swimming performance could be correlated with higher activity levels including a higher foraging rate, which would make them more prone to risky behaviors and increase the chances of reed isopods being detected by predators. A previous study on invertebrates which are subject to different predatory pressures from fish or dragonfly showed that in the absence of fish predation, less cautious behaviors might instead increase foraging activity [24]. The habitat-specific trade-offs detected in this study (Fig. 3) shows that is important not to infer trade-offs from comparisons of animals from different microenvironments, and trade-offs should always be quantified within similar ecological contexts [43].

These results suggest that predator-mediated selection has caused the ecotypes to diverge phenotypically. However, phenotypic plasticity could also have contributed to ecotype differentiation, and we have yet to determine to what extent traits such as shape or speed are heritable. Our results therefore clearly illustrate a case of rapid and parallel phenotypic divergence between two habitats, but not necessarily rapid evolution. The results do, however, suggest that predation might be a powerful diversifying force in this system, and that predator-mediated selection leads to a predictable outcome in the different lakes. Indeed, the deterministic force of selection has apparently an overriding effect over historical contingency [21]. We found no significant effects of lake origin or the interactions between lake and ecotype in any of the models for shape, head angle, escape speed or endurance (Fig. 2–3 and 5, Tables 1–4). This strong parallelism in antipredator adaptations is particularly remarkable in the light of how recent this divergence process is and strongly suggests that the changes we report here are adaptive [21]. Historical contingencies are known to influence evolutionary divergence [46]–[47], and are expected to exert their strongest effects during the early stages of adaptive diversification [48]. The rapidity of the ecotype divergence in these two lakes suggests that natural selection is strong and did quickly wipe out any possible signature of history in both lakes.

The integration of antipredator traits at the whole organism level can constrain adaptations [6], but also can be an outcome of selection and adaptation [25]. Genetic and phenotypic integration of traits with different functions can also result from correlational selection favoring different adaptive trait combinations in different habitats, morphs or ecotypes [25]. Previous studies have compared different populations and species with respect to their overall phenotypic integration patterns [25], [32] but have seldom if ever been unable to distinguish between ancestral and derived patterns of integration, especially in a parallel context. In our study, the reed isopods represent the original source phenotype, which makes it possible to infer that the original phenotypic trait combination was more modular than the derived one in the stonewort habitat (Fig. 6). We suggest that the higher level of phenotypic integration between morphology and pigmentation traits in the stonewort habitats is a result of predator-mediated correlational selection favoring increased integration in the novel habitat (Fig. 6).

In both Lake Tåkern and Lake Krankesjön, the reed ecotype has a higher modularity index, and shows a lower lever of phenotypic integration compared to the stonewort ecotype (Table 5). The reed ecotype has existed in both the lakes for a long time, in contrast to the more ephemeral stonewort habitat [28]. The more pronounced modular structure in the reed habitat (Table 5) might indicate that long-term stabilizing selection has favored a modular organization of the phenotypic traits [49]. In contrast, in the more recently formed stonewort ecotypes there are more edges between the pigmentation and the morphological traits in both lakes (Fig. 6, Table 5).

The adaptive significance of modularity in organismal evolvability has been discussed extensively [50]–[51]. In order to evolve new sets of traits which might belong to different phenotypic modules, selection might have needed to break down some of these modules [50]–[51]. Since overall prey crypsis is likely be a result of both pigmentation and morphological traits such as size and shape, it might be an adaptive response to the visually hunting fish predators in the stonewort habitat. Due to their low-resolution compound eyes, invertebrate predators have poor long-range vision [52], they do not entirely rely on visual cues to detect their prey and dragonfly larvae are typically sit-and-wait predators [8]. However, fish such as perch have high resolution vision and actively search and pursue their preys [30], [53], thus fish predation is likely to select for increased camouflage ability in the stonewort habitat. A previous study [18] has demonstrated locally differentiated pigmentation in different habitats and lakes over Sweden, as a result of selection for cryptic pigmentation. Moreover, predation from actively searching and fast-swimming predators like fish might also have selected for the lower overall behavioral activity [21] and slower speed (Figs. 2–3) in the stonewort habitat. Consequently, predator-mediated selection from fish in the stonewort habitat may have selected for novel trait combinations involving brighter pigmentation, prudent behaviors and lower speed to avoid the attention of predators. The overall higher level of phenotypic integration in the stonewort could be an adaptive phenotypic response to a new type of predator, which disrupted the ancestral modular phenotypic organization (Fig. 6).

The loss of a more modular phenotypic structure and a shift towards a higher level of phenotypic integration has, to our knowledge, not been found in previous studies of predation and its consequences on prey morphological adaptations. The loss of modularity might also have affected the overall body shape and decreased the swimming ability (Figs. 2–3). By disrupting the ancestral modular structure and increasing the overall level of phenotypic integration, predator-mediated selection might have resulted in a hydrodynamically less favorable shape in the stonewort habitat as a correlated response to selection for higher phenotypic integration [54]. This might indicate the existence of trade-off between higher overall morphological integration and the hydrodynamically most optimal shape in terms of swimming speed. There is thus a strong potential for conflicting selection pressures on different aspects of performance: correlational selection on size and pigmentation might have enhanced crypsis in the new habitat, whereas relaxed selection on escape speed might have decreased the hydrodynamic efficiency as a side-effect of selection for prudent behaviors. Indeed, although our data on exploratory behaviors comes from only one lake, and thus precludes generalization, we have also found in a previous study that stonewort individuals from both lakes had a lower propensity to engage mating, which also independently suggests a tendency to avoid exposure [21]. Selection for lower foraging activity and lower speed in turn might presumably result from the fact that isopods in the stonewort cannot escape from fish predators by swimming away, but have to rely on combinations of cryptic behavior, morphology and pigmentation to avoid predation.

In conclusion, here we have shown how different ecotypes have diverged in parallel in morphological and behavioral antipredator adaptations over a few decades and a few dozen generations. The picture that has emerged in this study is that these isopod ecotypes might have become adapted to different predation pressures in a multifactorial way, involving combinations and suites of morphological and behavioral traits. This study therefore adds to the increasing evidence that parallel evolution can be investigated even after few generations [3], from a single trait perspective to a multifunctional level, as rapid changes in overall phenotypic integration [54] might also arise rapidly.

## Supporting Information

Figure S1 Picture of a male A. aquaticus with the 10 landmarks used in the geometric morphometric analyses. One landmark was placed at the tip of the head, between both eyes, and then six landmarks were placed at both ends of the first, fourth and last thoracic segment, and then three landmarks at the end of the left, middle and right segment of the pleotelson.
(0.85 MB TIF)
