# Supplementary figures and images for: Contemporary Parallel Diversification, Antipredator Adaptations and Phenotypic Integration in an Aquatic Isopod
Source: PLoS One. 2009 Jul 9;4(7):e6173. doi: 10.1371/journal.pone.0006173 (PMC2704376; doi:10.1371/journal.pone.0006173)

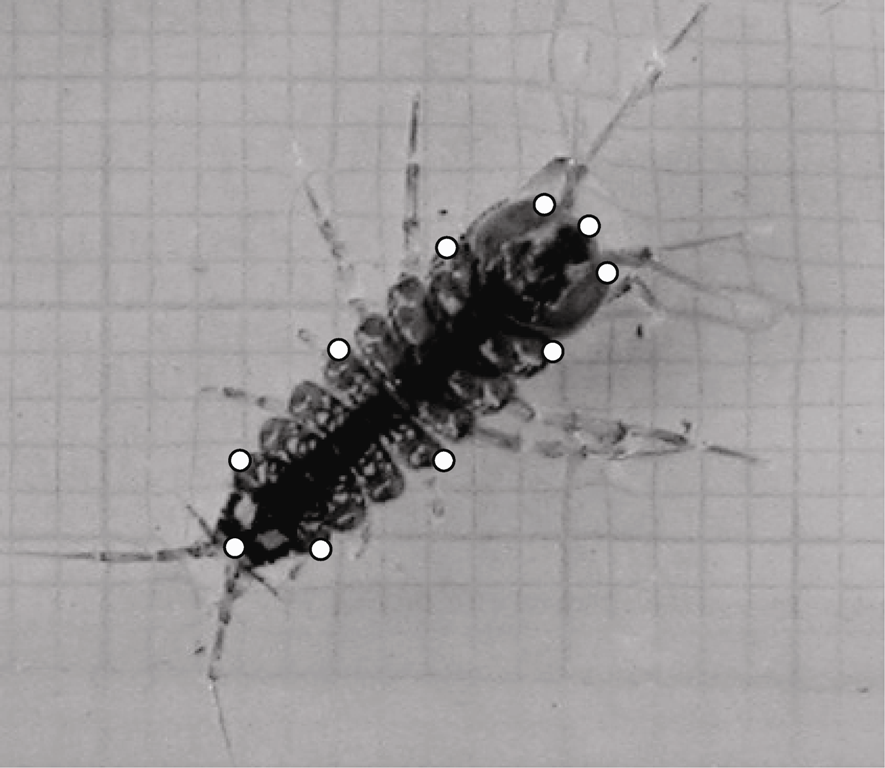

Supplement: Figure S1 — Picture of a male A. aquaticus with the 10 landmarks used in the geometric morphometric analyses. One landmark was placed at the tip of the head, between both eyes, and then six landmarks were placed at both ends of the first, fourth and last thoracic segment, and then three landmarks at the end of the left, middle and right segment of the pleotelson. (0.85 MB TIF) [file pone.0006173.s001.tif]
